# Promising Low-Cost Adsorbent from Waste Green Tea Leaves for Phenol Removal in Aqueous Solution

**DOI:** 10.3390/ijerph19116396

**Published:** 2022-05-24

**Authors:** Asmat Ali, Maria Siddique, Wei Chen, Zhixin Han, Romana Khan, Muhammad Bilal, Ummara Waheed, Irum Shahzadi

**Affiliations:** 1 School of Environmental Studies, China University of Geosciences, Wuhan 430078, China; asmat@cug.edu.cn; 2 State Key Laboratory of Biogeology and Environmental Geology, China University of Geosciences, Wuhan 430078, China; 3 Hubei Key Laboratory of Environmental Water Science in the Yangtze River Basin, China University of Geosciences, Wuhan 430078, China; 4 Department of Environmental Sciences, COMSATS University Islamabad, Abbottabad Campus, Abbottabad 22060, Pakistan; romanakhan@cuiatd.edu.pk (R.K.); mbilal@cuiatd.edu.pk (M.B.); 5 Geological Exploration Institute of Shandong Zhengyuan, China Metallurgical Geology Bureau, Tai’an 271000, China; rroobbeerrtt1990@163.com; 6 Institute of Plant Breeding and Biotechnology, MNS University of Agriculture, Multan 59300, Pakistan; ummara.waheed@mnsuam.edu.pk; 7 Department of Biotechnology, COMSATS University Islamabad, Abbottabad Campus, Abbottabad 22060, Pakistan; irumayaz@cuiatd.edu.pk

**Keywords:** wastewater treatment, phenol, waste green tea leaves (WGTLs), low-cost adsorbent, sono-adsorption

## Abstract

Phenol is the most common organic pollutant in many industrial wastewaters that may pose a health risk to humans due to its widespread application as industrial ingredients and additives. In this study, waste green tea leaves (WGTLs) were modified through chemical activation/carbonization and used as an adsorbent in the presence of ultrasound (cavitation) to eliminate phenol in the aqueous solution. Different treatments, such as cavitation, adsorption, and sono-adsorption were investigated to remove the phenol. The scanning electron microscope (SEM) morphology of the adsorbent revealed that the structure of WGTLs was porous before phenol was adsorbed. A Fourier Transform Infrared (FTIR) analysis showed an open chain of carboxylic acids after the sono-adsorption process. The results revealed that the sono-adsorption process is more efficient with enhanced removal percentages than individual processes. A maximum phenol removal of 92% was obtained using the sono-adsorption process under an optimal set of operating parameters, such as pH 3.5, 25 mg L^−1^ phenol concentration, 800 mg L^−1^ adsorbent dosage, 60 min time interval, 30 ± 2 °C temperature, and 80 W cavitation power. Removal of chemical oxygen demand (COD) and total organic carbon (TOC) reached 85% and 53%. The Freundlich isotherm model with a larger correlation coefficient (R^2^, 0.972) was better fitted for nonlinear regression than the Langmuir model, and the sono-adsorption process confirmed the pseudo-second-order reaction kinetics. The findings indicated that WGTLs in the presence of a cavitation effect prove to be a promising candidate for reducing phenol from the aqueous environment.

## 1. Introduction

Many industrial processes produce large amounts of wastewater that is polluted by various organic and inorganic substances. Due to their industrial importance, phenolic compounds (PCs) are among the most common organic pollutants in many industrial wastewaters [1]. At present, the worldwide production rate of phenol is about 6 million tons per year, and it is showing a significant growth trend [1,2]. Phenol and its derivatives are no less than a menace to global human health and natural ecosystems [3,4,5]. These pollutants reduce the light that can reach photosynthetic organisms and change riverbed characteristics, making it an unsuitable habitat for many invertebrates [6]. Among various similar pollutants, phenol is extensively used as a reactive agent, solvent, refrigerant, and in different manufacturing processes. It is found in the industrial discharge of resin, plastics, explosives, pharmaceutical, steel, leather, etc. [7]. The presence of phenol in wastewater can result in ecosystem changes, health problems, and surface water contamination. Excessive inhalation or exposure to phenol can cause coma, convulsions, cyanosis, and other adverse reactions [8]. Phenol has been listed as one of the hazardous pollutants by the United States Environmental Protection Agency (USEPA) which has restricted its use to up to 1 mg L^−1^ for sewerage water [9]. Chronic exposure to high phenol concentration results in protein precipitation and causes cell damage [10,11]. Even exposure to low concentrations of phenol can result in protein denaturation and cause impaired vision, cancer, diarrhea, and dark urine excretion [12].

Therefore, phenol removal from wastewater has become a dire need before wastewater discharges. Different treatment techniques have been used for phenol removal, from polluted water [6] and wastewater. These include electrochemical oxidation, adsorption by carbon fibers or activated carbon, waste material, wet air oxidation, chemical coagulation, solvent extraction, membrane separation, and photocatalytic degradation [13,14,15].

Among the above-mentioned treatments, adsorption has been recognized as a highly effective treatment due to its easy operation and potential for application on a large scale. Several adsorbents have been used to date, such as biomasses [16], polymers [17], rice husk [11], polymer composites [18], and activated carbon [19]. Activated carbon (AC) as an effective adsorbent has been widely utilized. Still, its monetary cost makes it less attractive due to its complex regeneration, despite its effectiveness and efficiency [20]. Lately, with the rise in cost and scarcity of raw materials, it is imperative to explore low-cost alternatives [21]. For this reason, agricultural trash has been the focus of most researchers. The by-products of agricultural trash lack carbon-based substances and serve as a breeding ground for bacteria. Its substandard disposal results in it becoming a menace to the environment. Moreover, it is a hard decision to lessen the meager agricultural profit through the cost of waste management, and therefore the use of waste materials should be explored [22].

Green tea, scientifically known as *Camellia sinensis*, is a popular beverage worldwide. The unfermented buds and leaves of this plant are pale color and bitter to taste. In some Asian countries, such as Pakistan, tea is one of the most consumed beverages, second only to water. After its consumption, it is abundantly available as household waste. It is discarded after being used as the raw material in producing drinks, health foods, nutritional s, and cosmetic products. Waste green tea leaves (WGTLs) are found to be natural adsorbents that can remove toxic pollutants [23].

The adsorption quality of waste green tea leaves can be boosted through activation and hybrid techniques for the complete mineralization of contaminants. The advanced oxidation process (AOP) effectively degrades organic contaminants due to highly reactive ˙OH (hydroxyl radical) production in the aqueous medium without a catalyst. Therefore, cavitational phenomena is one of the most dominant and fastest-growing research fields regarding the broad applications for water and the wastewater treatment of pollutants [24]. Acoustic cavitation involves generating and growing tiny bubbles that collapse suddenly because of their interaction with sound energy. This causes bubbles to dissolve in the liquid medium due to higher temperature and pressure conditions within the dissolving bubbles [25]. This results in physical and chemical effects within the solution that aid in the degradation of organic pollutants such as dyes, phenol, etc. [26]. Cavitation in the AOP to degrade phenol has been found efficient and economically workable [27,28]. The present study aims to examine the removal efficiency of cavitation, adsorption, and sono-adsorption for phenol by using waste green tea leaves (WGTLs) to function certain factors such as pH and contact time adsorbent dosage, adsorbate concentration, and the ultrasonic power in aqueous solution. Moreover, thermodynamics, kinetics, and adsorption isotherms have been explored to understand the process mechanism.

## 2. Materials and Method

### 2.1. Reagents Preparations

Phenol (CAS. No.108-95-2, purchased from Sigma-Aldrich, Taufkirchen, Germany) was used as a model organic pollutant. Sodium hydroxide (NaOH), hydrochloric acid (HCl, 37%), phosphoric acid (H_3_PO_4_, 85%), and sulfuric acid (H_2_SO_4_, 95–97%) were purchased at the highest available purity level (Sigma-Aldrich, Taufkirchen, Germany and Merck, Darmstad, Germany). These chemicals were used to analyze the chemical oxygen demand (COD), total organic carbon (TOC), and pH adjustment of the samples. Green tea from commercial tea bags (Lipton brand) was used to obtain WGTLs. A 1000 mg L^−1^ stock solution was prepared with a specific concentration of phenol that was further dissolved in distilled water (1000 mL), while working concentrations were prepared by appropriate dilution from the stock.

### 2.2. Preparation of Adsorbent from Waste Green Tea Leaves (WGTLs)

Tea infusions were first prepared by steeping some tea bags in tap water (125 mL of water per tea bag) at 90 °C for 3 min. After that, WGTLs were recovered and washed with distilled water (ca. 375 mL) until the transparent filtrate appeared. The material was then rinsed, and oven dried at 60 °C for 24 h. Finally, the WGTLs were ground and sieved to particles < 500 µm. The chemical activation of WGTLs was carried out by employing the material in H_3_PO_4_ (2.5 M) for 24 h. (1:3 ratio). Subsequently, the material was positioned in a furnace at 400 °C for 4 h. (under nitrogen condition). Finally, the prepared, treated material (adsorbent) was washed with deionized water (DI) till pH at 7 and oven-dried for 24 h at 110 °C. The adsorbent was then stored in plastic bags for further experiments.

### 2.3. Analytical Procedure for the Experimentation

Aqueous samples were investigated for their pH through a digital pH meter (PHS-38W Microprocessor, Tsingtao Toky Instruments Co., Ltd., Qingdao, China). A UV/Vis. spectrophotometer (T80 + PG Instruments, Lutterworth, UK) was used to determine phenol concentration at the wavelength of 269 nm. An ultrasonic bath (Cleaner model U Tech Products, Inc., Schenectady, NY, USA) was calibrated as reported [29] and used for the cavitation and sono-adsorption processes experiments. COD was analyzed after sample digestion (through the COD digester model (TR320, Merck Spectroquant)). The TOC was determined by injecting a 20 mL sample into the TOC analyzer (Shimadzu, model TOC-V CSH) in the furnace at 680 °C. The surface morphology of the WGTLs was investigated by a scanning electron microscope (SEM) instrument (Model-JSM 5910, JEOL, Tokyo, Japan). The Fourier Transform Infrared Spectroscopy (FTIR) analysis was performed, and IR spectra were recorded using the Potassium bromide (KBr) pellet technique on a FTIR spectrometer (Model-Perkin Elmer Spectrum 10D, Waltham, MA, USA) with a spectrum range of 500–4000 cm^−1^ using a Resolution of 0.5 cm^−1^.

### 2.4. Experiments

Cavitation, adsorption, and sono-adsorption experimental sets were adopted for the removal of phenol. Sono-adsorption and cavitation experiments were performed using a conical flask that was immersed in an ultrasonic bath (35 kHz). For sample pH adjustment, NaOH and HCl (1 M) were used. Blank and control experiments were conducted in every set of the experiment to prevent possible contamination or change during the cavitation, adsorption, and sono-adsorption experiments. All experiments were performed in triplicates.

#### 2.4.1. Cavitation Experiment

The cavitation experiments were performed using 50 mL working volume with operating parameters including pH, contact time, ultrasonic frequency, and pollutant concentration. The continuous water circulation maintained the temperature (30 ± 2 °C) to control the internal temperature of the experiments in an ultrasonic bath. In the cavitation’s experiments, the ultrasound (US) power was adjusted at 80 W for cavitation by keeping the other conditions constant, i.e., 3.5 pH, 25 mg L^−1^ initial phenol concentration, 0–180 min reaction time at a temperature of 30 ± 2 °C. The optimized operating parameters from the cavitation and adsorption experiments were taken to test the adsorption process.

#### 2.4.2. Adsorption Experiment

The batch adsorption experiments were performed in a 250 mL conical flask using a known amount of adsorbent with the optimum conditions of other operating parameters and positioned in an orbital shaker at 30 ± 2 °C (220 rpm). The adsorption experiments were conducted at 3.5 pH, 25 mg L^−1^ initial phenol concentration, 800 mg L^−1^ adsorbent dose, and 0–180 min reaction time at a temperature = 30 ± 2 °C. After attaining the preferred contact time, samples were taken, filtered using a 40-micron filter paper and analyzed using UV/Vis. Spectrophotometer.

#### 2.4.3. Sono-Adsorption Experiment

To explore the effect of the sono-adsorption process for phenol removal, experiments were conducted using a conical flask in an ultrasonic bath. The experiments were carried out using optimized operating parameters i.e., 25 mg L^−1^ of phenol concentration, 3.5 pH, 60 min contact time, 800 mg L^−1^ adsorbent dose, ultrasonic frequency of 35 kHz, at 30 ± 2 °C temperature, and 80 W ultrasound (US) power for cavitation. After 10 min, a 3 mL sample was obtained without disturbing the reaction mixture. The samples were filtered and analyzed by a UV/Vis. Spectrophotometer.

#### 2.4.4. Calculation of Phenol Removal Efficiency

The residual phenol concentration was measured using a UV/Vis. spectrophotometer. Equation (1) was used to calculate the removal efficiency (R, %) of phenol:

(1) $$R=\frac{C_{i}-C_{t}}{C_{i}}\times 100$$

where C*_i_* is the phenol initial conc. (mg L^−1^), and C*_t_* is conc. (mg L^−1^) at time *t* during the experiment. Similarly, TOC and COD removal percentage (R_TOC_ and R_COD_, %) of the samples was measured using Equations (2) and (3), respectively:

(2) $$R_{\mathrm{TOC}}=\frac{{\mathrm{TOC}}_{i}-{\mathrm{TOC}}_{t}}{{\mathrm{TOC}}_{i}}\times 100\%$$

(3) $$R_{\mathrm{COD}\;}=\frac{{\mathrm{COD}}_{i}-{\mathrm{COD}}_{t}}{{\mathrm{COD}}_{i}}\times 100\%$$

where TOC*_i_* and COD*_i_* is the TOC and COD initial conc. (mg L^−1^), respectively; and TOC*_t_* and COD*_t_* is TOC and COD conc. (mg L^−1^) at time *t*, respectively.

#### 2.4.5. Adsorption Isotherms and Kinetics Analysis

The initial phenol concentrations (5–100 mg L^−1^) were used for the adsorption isotherms experiments. The conditions of operating parameters, i.e., adsorbent dose (800 mg L^−1^), rotation speed (220 rpm) and temperature (30 ± 2 °C), pH (3.5) were kept constant for the interpretation of adsorption isotherm models. The following Equations (4) and (5) for the Langmuir isotherm and Freundlich models were applied to describe the phenol adsorption by WGTLs, respectively:

(4) $$q_{e}=\frac{\left(K_{ads}q_{max}C_{e}\right)}{\left(1+K_{ads}C_{e}\right)}$$

(5) $$q_{e}=K_{f}C_{e}^{1/n}$$

where q*_e_* (mg g^−1^) indicates the quantity of phenol adsorbed at equilibrium, q*_max_* (mg g^−1^) is the maximum adsorption capability, K*_ads_* is the equilibrium constant, C*_e_* is the adsorbate phenol concentration (mg L^−1^) in solution, K*_f_* is the Freundlich isotherm constant, *n* represents the heterogeneity of the WGTLs.

Kinetics studies were performed using the initial phenol concentration of phenol, i.e., 10, 25, 50, 100 mg L^−1^ by keeping constant the other operating conditions at variable contact times. The data obtained were analyzed using pseudo-first-order and pseudo-second-order kinetic models. The sorption kinetics are essential in evaluating the sorption efficiency and exploring the mechanism and rate of phenol adsorption onto the surface of WGTLs. The pseudo-first-order and pseudo-second-order kinetic models were expressed as follows in Equations (6) and (7):

(6) $$q_{t}=q_{e}\left(1-e^{-k_{1}t}\right)$$

(7) $$q_{t}=\frac{k_{2}q_{e}^{2}t}{1+k_{2}q_{e}t}$$

where q*_t_* (mg g^−1^) is the amount of adsorbed phenol at time *t*, k_1_ (min^−1^) and k_2_ (min^−1^) are the pseudo-first-order and pseudo-second-order rate constants, respectively [30,31].

The OriginPro software (version 2021, OriginLab) was employed to fit the data for both isotherm and kinetics models using the nonlinear fitting analysis function.

## 3. Results and Discussion

### 3.1. Characterization of Adsorbent (WGTLs)

#### 3.1.1. Scanning Electron Microscope (SEM) Analysis

**Figure 1:**
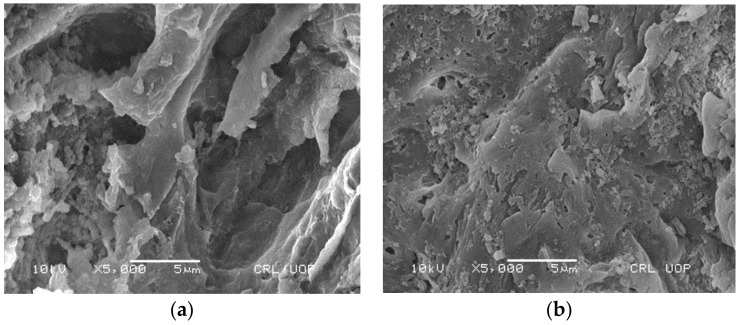
SEM image of WGTLs before (**a**) and after (**b**) sono-adsorption process.

The scanning electron microscope (SEM) analysis was performed for investigating the surface morphology of the adsorbent. The pre-and post-treated SEM metaphors of waste green tea leaves are shown in Figure 1 for the sono-adsorption process. The SEM images of WGTLs that are shown in Figure 1a reveal that before sono-adsorption, the adsorbents had rough surfaces containing pores of different sizes. The changeable pore size ensured the adsorption of phenol molecules on the surface of the WGTLs where an appropriate surface medium was provided. Figure 1b verified that adsorbate molecules filled the cavities’ surfaces. Similar morphology was reported in the earlier studies [32,33].

#### 3.1.2. Fourier Transform Infrared Spectroscopy (FTIR) Study

**Figure 2:**
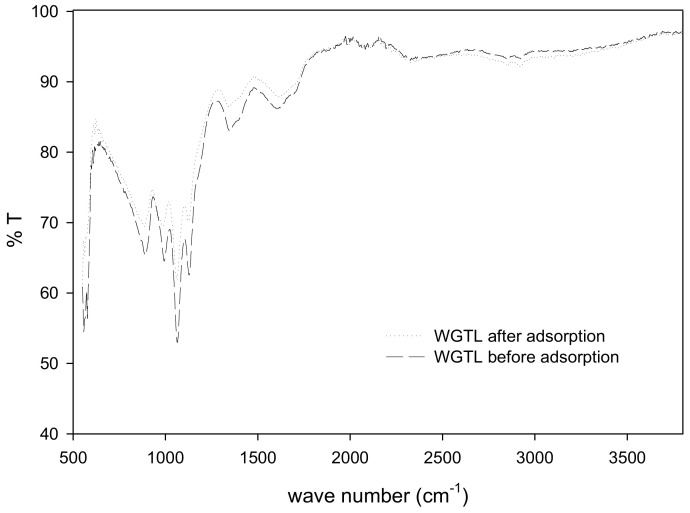
Fourier Transform Infrared Spectroscopy (FTIR) spectra of phenol and WGTLs (before and after adsorption).

Infrared (IR) spectra for WGTLs before and after adsorption of phenol were obtained in 500–3500 cm^−1^ Figure 2. The FTIR spectra of WGTLs before phenol adsorption do not display the peaks at ~3400 cm^−1^ and ~2950–2900 cm^−1^ due to the structural and chemical modification of waste green tea leaves by chemical activation and carbonization process. The FTIR spectra of WGTLs after phenol adsorption shows some changes in the bands [34]. Moreover, the corresponding bands at 2956 and 2923 cm^−1^ represent the O–H stretching. A band at 1667 cm^−1^ was observed, meaning a C=O stretch of keto groups that show phenol degradation [35,36].

### 3.2. Factors Influencing the Cavitation and Adsorption Processes

#### 3.2.1. Contact Time

**Figure 3:**
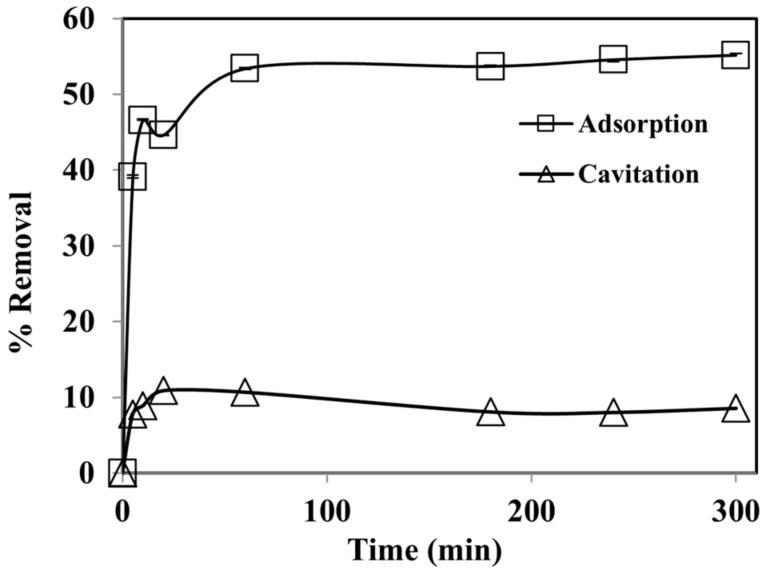
The effect of contact time on phenol removal by adsorption and cavitation processes. Experimental conditions: phenol conc. = 25 mg L^−1^, pH = 3.5; cavitation power = 80 W; adsorbent dose = 800 mg L^−1^; temperature = 30 ± 2 °C.

Figure 3 represents the efficacy of contact timings on the percentage of phenol removal using cavitation and adsorption processes ranges (0–180 min). It was noted that phenol removal was at a maximum initially up to a contact time of 60 min in both processes. A value of 53% of phenol removal efficiency was attained by adsorption process using WGTLs and the percentage removal of phenol by ultrasonic cavitation yielded a removal efficacy of 10%. Due to the physical and chemical properties, phenol is expected to remain more in bulk than the vapor phase throughout the cavitation process [37]. The low removal efficiency of phenol was observed in the bulk form where hydroxyl radicals were produced, recombined, and thus were unavailable to outbreak the phenol molecules. Additionally, after the initial cavitation period, whatever dissipated gas in the solution is present is vented, resulting in the reduced initiation of ˙OH radicals, as explained in the previous research [38]. While in adsorption, the maximum sorption of phenol was attained up to 60 min, as shown in Figure 2. Optimum sorption of the phenol was achieved, and the rate of sorption was higher than the rate of desorption. The same trend was noted in earlier research where after 60 min the equilibrium was established due to the capacity level of effective sites over the surface of WGTLs [39,40].

#### 3.2.2. pH

**Figure 4:**
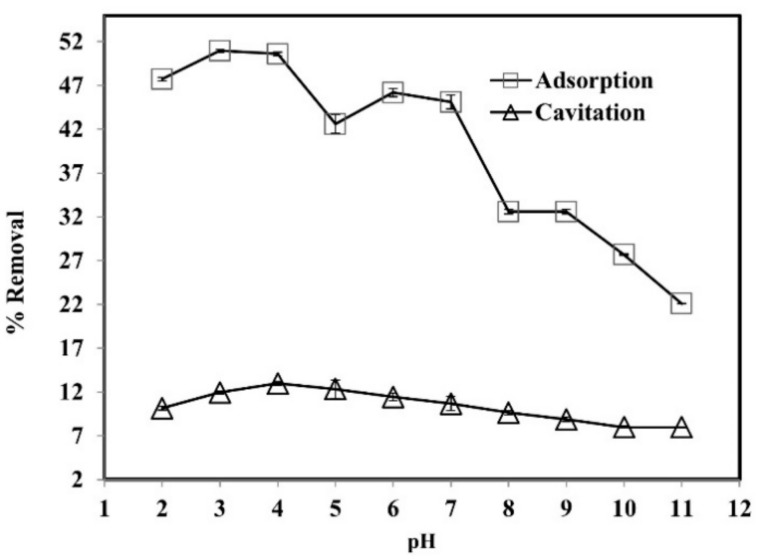
The effect of pH on the removal of phenol by adsorption and cavitation processes. Experimental conditions: phenol conc. = 25 mg L^−1^; adsorbent dose = 800 mg L^−1^; cavitation power = 80 W; temperature = 30 ± 2 °C; time = 60 min.

pH is considered as the key parameter for adsorption experiments. The effect of variable pH ranges (2–11) was investigated on phenol removal, as demonstrated in Figure 4. The removal of phenol was enhanced in acidic conditions and reduced with the increase in solution pH. Consequently, the cavitation experiments were conducted at an acidic pH.

The acid dissociation constant (p*K*_a_) of phenol in water is 9.95. At a lower pH than p*K*_a_, the phenolic molecules may enter the water bubble and thermally decompose with **˙**OH in the outer bubble [41]. On low pH values, the adsorbent materials surface is positively charged. Under alkaline conditions, it has a negative charge. In the acidic situation of pH 2–6 there is a powerful attraction among the positive surface adsorbent and a phenol molecule. When the pH of the solution is more than p*K*_a_, then phenol dissociates into phenolate ions. A repulsion force exists between the negatively charged carbon surface and phenolate ions, resulting in decreased absorption. Therefore, the adsorption process removed a substantial quantity (51%) of phenol. While at pH 11 only 22% phenol removal was observed when the pH of the solution was above seven, so the dissociated phenolate results in the repulsion from the negative charge from the surface of the activated the adsorbent [42].

#### 3.2.3. Initial Phenol Concentration

**Figure 5:**
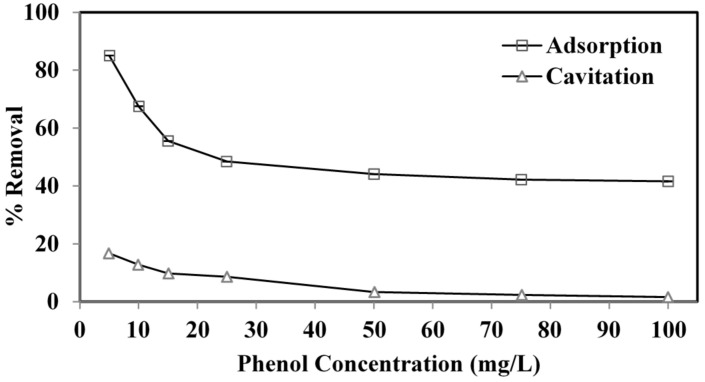
Effect of initial phenol concentration on removal of phenol by adsorption and cavitation processes. Experimental conditions: pH = 3.5, adsorbent dose = 800 mg L^−1^, cavitation power = 80 W, temperature = 30 ± 2 °C, time = 60 min.

The effect of the initial phenol content was studied using cavitation and adsorption techniques. Figure 5 shows that increasing the initial phenol concentration from 5–100 mg L^−1^ reduced the phenol removal effectiveness from 17 to 1.5% and from 85 to 41.5% in the cavitation and adsorption processes. Deficient active adsorbent surfaces contribute to reduced removal effectiveness, which leads to increased phenol content in the mass solution [40]. A possible explanation for the cavitation process is that increased concentration forms a complex hydrogen (H) bond between phenolic compounds. Higher phenol concentrations increase phenol’s molecule number but not hydroxyl radicals, resulting in a slower elimination rate. Because of the H bonds between nearby molecules, molecules with carboxyl (COOH) or carbonyl (CHO) groups can exist as a compound in the solution. This complex can also block their transfer against the bubble interaction, decreasing phenol degradation [29,40,43].

#### 3.2.4. Effect of Ultrasound (US) Power

**Figure 6:**
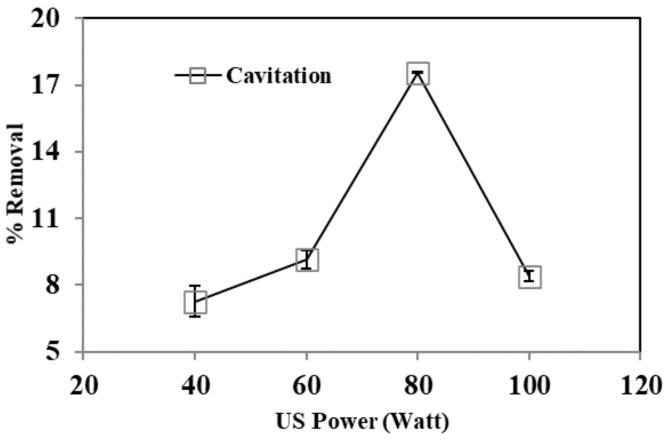
The effect of ultrasonic power on cavitation process. Experimental conditions: phenol conc. = 25 mg L^−1^, pH = 3.5, temperature = 30 ± 2 °C, time = 60 min.

US power is an essential factor influencing the cavitation action and consequently, the degradation processes. Various US power experiments (40–100 W) were conducted for optimization. At low power, the vibration amplitude is insufficient to provide the requisite pressure differential allowing cavitation bubbles to form. Figure 6 shows that the removal effectiveness increased from 40 to 80 W, and that after 60 min, the maximum phenol removal was achieved at 80 W. As power increases, more cavitation bubbles form as different nucleation sites reach their limit. The rise in US power may also increase mixing intensity due to the instability created by the cavitation bubbles collision. However, it was found that an increase in US power from 80 to 100 W could not enhance phenol removal after 60 min. When power is increased significantly over the cavitation limit, cavitation intensity levels off. A cavitation effect is slightly self-limiting. After all, the current ultrasonic bubbles prevent the effective sound-wave transmission required to create more ultrasonic bubbles. Further, when a chemical reaction begins, the acoustic power is optimal for a maximal reaction rate. High US power generates more bubbles, obstructing the flow of acoustic energy [29,44].

#### 3.2.5. Effect of Adsorbent Dosage

**Figure 7:**
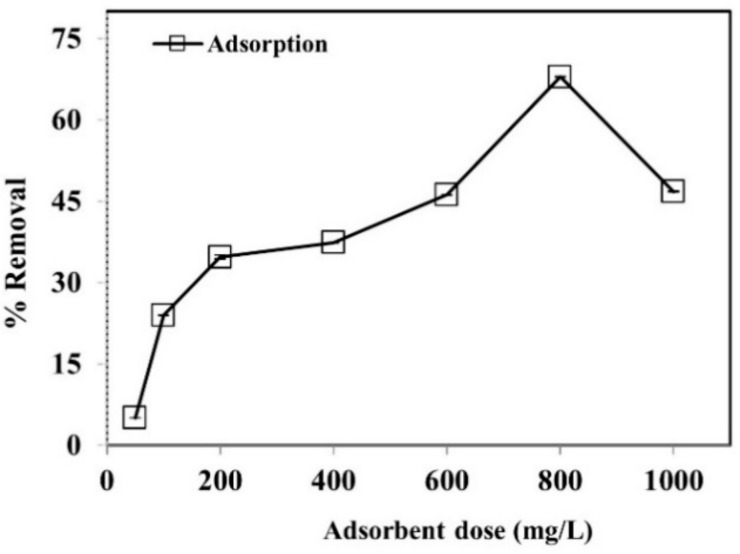
The effect of adsorbent dosage on phenol removal using adsorption process. Experimental conditions: phenol conc. = 25 mg L^−1^, pH = 3.5, temperature = 30 ± 2 °C, time = 60 min.

The adsorbent dose is a vital factor in the adsorptive process and is dependent on the available adsorbent sites. The efficiency of different adsorbent doses on phenol removal was studied under optimum conditions as shown in Figure 7. The results found that the increase in the adsorbent concentration increased the percentage adsorption of phenol to a certain level, and beyond that the adsorption decreases. It is evident that during the initial 60 min of the experiment, the phenol removal was at a maximum because of the availability of substantial binding spots on the surface of WGTLs. In addition, nearly 67% phenol elimination was accomplished under the best adsorbent dosage of WGTLs (800 mg L^−1^). WGTLs were modified by the chemical activation/carbonization process which provided much porosity and a high surface area to the adsorbent material, [45,46] enhancing the adsorption process. These findings were reported in an earlier published report [7].

### 3.3. Sono-Adsorption Process

**Figure 8:**
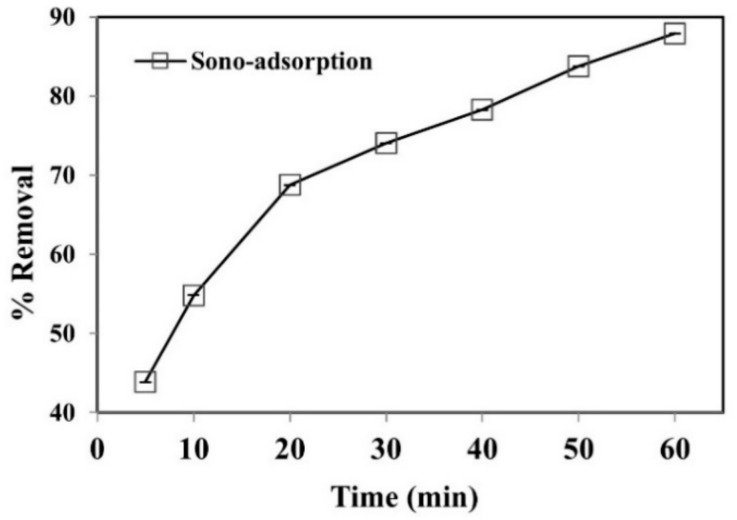
Removal of phenol using sono-adsorption by WGTLs. Reaction conditions: time = 60 min, phenol conc. = 25 mg L^−1^, pH = 3.5, adsorbent dose = 800 mg L^−1^, cavitation power = 80 W, temperature = 30 ± 2 °C.

Further experiments were carried out to determine the synergistic mechanism of the coupled sono-adsorption process to remove phenol. The sono-adsorption removal efficacy of phenol was found to be higher than adsorption and cavitation alone (Figure 8). The results show that up to 92% of phenol was successfully removed under optimum conditions. Similar results were documented in previous reports [47,48]. The cavitation mechanism is the nucleation, expansion, and collapse of the transient gas bubbles driven by an ultrasound wave in a liquid medium. The convection creates physical phenomena such as micro-streaming, micro-turbulence, acoustic (or shock) waves, and micro-jets. An ultrasonic enactment gives rise to cavitation in the medium. Due to the compression and rarefaction, microbubbles were developed in the medium. The quick bubble collapse results in a generation of extremely high temperatures (5000 K) and pressures (500 bar) inside the bubble [49]. The development of temperature extremes and pressures inside the bubbles creates chemical and physical effects, i.e., causing shock waves and reactive free radicals (e.g., **˙**OH, HO_2_, and O**˙**) [50,51]. Dissolution of the bubble at the time of the maximum compression releases these radicals in the medium. They speed up and accelerate chemical reactions known as sonochemical reactions [52].

### 3.4. Total Organic Carbon and Chemical Oxygen Demand Reduction

**Figure 9:**
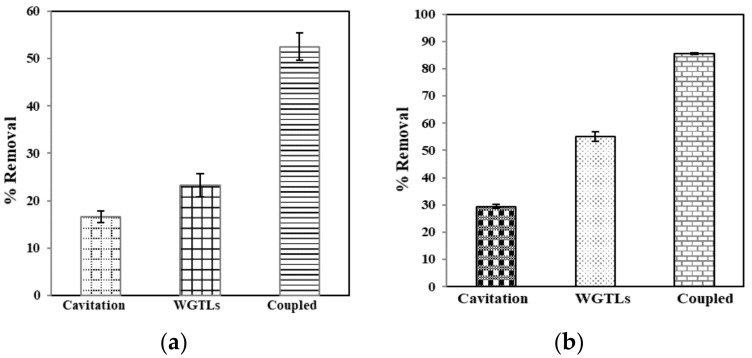
Removal of TOC (**a**) and COD (**b**) by cavitation, adsorption (WGTLs) and sono-adsorption (coupled) processes.

The results revealed that TOC and COD removal in the sono-adsorption process was improved in comparison to the individual processes, i.e., cavitation and adsorption, as shown in Figure 9. The decrease in the TOC and COD concentrations confirms the removal of phenol [53]. The coupled method for TOC and COD removal was found to be comparatively additional. It suggests that cavitation has an enormous collaborative effect on removing organic pollutants (phenol) by the WGTLs in the adsorption process. The breakdown of the cavitation bubbles could cause a sizeable mechanical pressure on the surface of the adsorbent, as described by previous reports [54,55].

### 3.5. Adsorption Isotherms

This study evaluated the interactive character of phenol in adsorption by WGTLs by the most widely used isotherm models, i.e., Langmuir and Freundlich isotherms [56]. Langmuir’s model defined similar, single-layer, and equal energies over adsorption sites. While the Freundlich model explains reversible multi-layer adsorptive fixation to heterogeneous adsorbent materials. The maximum possible adsorption capacity was achieved at equilibrium without reciprocal interaction between the phenol molecules. Once the phenol molecule consumes an empty site, additional attachments are restricted because of the chemical bond formation. The Freundlich model confirms the pollutant adsorption as multi-layers on the adsorbent’s surface [55,56].

**Figure 10:**
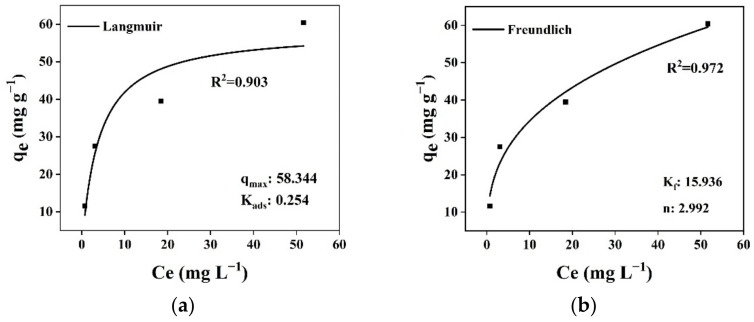
Nonlinear data fitting for Langmuir (**a**) and Freundlich (**b**) models for isothermal adsorption of WGTLs.

Figure 10 shows that data are fitted to both isotherm models in the adsorption of phenol by WGTLs using the nonlinear regression. The Freundlich isotherm illustrated multi-layer adsorption with a higher coefficient of correlation (R^2^) value (0.972) while comparing the Langmuir model with a deprived R^2^ value (0.903). This indicated multilayer reversible attachment of the adsorbate over heterogeneous WGTLs. Furthermore, the Freundlich constant K*_f_* was used as an indicator of adsorption capacity. Thus, the large value of K*_f_* showed the high adsorption capacity of phenol on WGTLs. Values of the 1/n were observed to be less than 1, also suggesting the existence of physisorption between phenol and WGTLs.

### 3.6. Adsorption Kinetics

**Figure 11:**
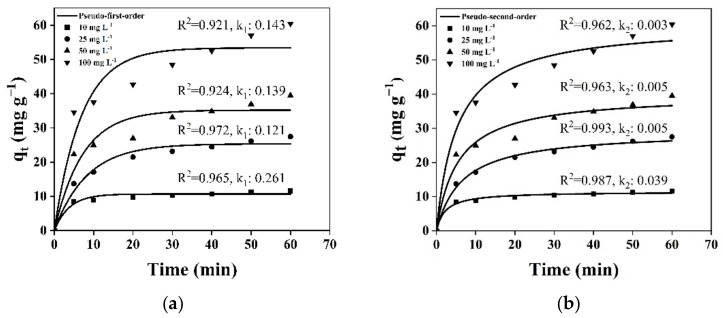
Nonlinear fitting for pseudo-first-order (**a**) and pseudo-second-order (**b**) models to phenol removal by WGTLs at various initial concentrations.

A kinetics study is a critical consideration to find out the adsorption potential of the adsorbent. The pseudo-first-order and pseudo-second-order kinetic models were utilized to understand the adsorption kinetics of phenol onto WGTLs. Adsorption is a two-step process including the transportation of the adsorbate molecules from the bulk solution into the solid phase, followed by dispersion in the interior of the pores [57]. The best fitted pseudo-second-order kinetics models assume that chemisorption is responsible for controlling the phenol adsorption and entails the substitution/distribution of electrons between WGTLs and phenol. Figure 11 shows the rate constants (k_1_ and k_2_) and coefficients of correlation (R^2^) of both the pseudo-first-order and pseudo-second-order models at variable initial phenol concentrations using the non-linear fitting. It reflected the better fit to phenol adsorption by WGTLs using the pseudo-second-order model (R^2^, 0.962–0.993) than the pseudo-first-order model (R^2^, 0.921–0.972). This indicated that chemisorption is largely involved in the removal of phenol by WGTLs. However, a decrease in k_2_ was observed (0.039, 0.005, 0.005 and 0.003 min^−1^) with increasing phenol concentrations, from 10, 25, 50 to 100 mg L^−1^, respectively, which showed that chemisorption is not the sole adsorption-limiting factor. Therefore, in addition to chemisorption, physisorption drives the adsorption process. Furthermore, simultaneous chemisorption and physisorption may work in combination for heterogeneous adsorption in this research work.

## 4. Conclusions

The current study demonstrates that waste green tea leaves (WGTLs) can be used as a potential adsorbent for the removal of phenol from aqueous solutions. The sono-adsorption method was found to have a higher potential for phenol removal than other techniques such as cavitation and adsorption processes. The maximum removal efficiency was 92% in 60 min along with the optimization of influential parameters, i.e., 3.5 pH, 800 mg L^−1^ adsorbent dose, 80 W ultrasonic power, and 30 °C temperature. TOC removal was 85% and COD removal was 53%, indicating phenol removal. Multi-layer adsorption and chemisorption could be the major process for the heterogeneous adsorption of phenol on WGTLs, but physisorption also occurred. The findings indicated that WGTLs in the presence of a cavitation effect proved to be promising candidates for reducing phenol from the aqueous environment in developing countries in Asia.

## Data Availability

The data that support the findings of this study are available from the corresponding author, Maria Siddique (maria@cuiatd.edu.pk) upon reasonable request.
